# S100A8/A9 Is a Marker for the Release of Neutrophil Extracellular Traps and Induces Neutrophil Activation

**DOI:** 10.3390/cells11020236

**Published:** 2022-01-11

**Authors:** Evelien G. G. Sprenkeler, Judith Zandstra, Nadine D. van Kleef, Ines Goetschalckx, Bibian Verstegen, Cathelijn E. M. Aarts, Hans Janssen, Anton T. J. Tool, Gerard van Mierlo, Robin van Bruggen, Ilse Jongerius, Taco W. Kuijpers

**Affiliations:** 1 Sanquin Research and Laboratory Services and Landsteiner Laboratory, Department of Molecular Hematology, Amsterdam University Medical Center (AUMC), University of Amsterdam, 1066 CX Amsterdam, The Netherlands; nadinevankleef23@gmail.com (N.D.v.K.); I.Goetschalckx@sanquin.nl (I.G.); bibianverstegen@gmail.com (B.V.); c.aarts@sanquin.nl (C.E.M.A.); a.tool@sanquin.nl (A.T.J.T.); r.vanbruggen@sanquin.nl (R.v.B.); 2 Department of Pediatric Immunology, Rheumatology and Infectious Diseases, Emma Children’s Hospital, AUMC, University of Amsterdam, 1105 AZ Amsterdam, The Netherlands; j.zandstra@sanquin.nl (J.Z.); I.Jongerius@sanquin.nl (I.J.); 3 Sanquin Research and Laboratory Services and Landsteiner Laboratory, Department of Immunopathology, AUMC, University of Amsterdam, 1066 CX Amsterdam, The Netherlands; g.vanmierlo@sanquin.nl; 4 Division of Biochemistry, The Netherlands Cancer Institute, 1066 CX Amsterdam, The Netherlands; j.janssen@nki.nl

**Keywords:** neutrophils, neutrophil extracellular traps, S100A8/A9, MRP8/14, calprotectin

## Abstract

Neutrophils are the most abundant innate immune cells in the circulation and they are the first cells recruited to sites of infection or inflammation. Almost half of the intracellular protein content in neutrophils consists of S100A8 and S100A9, though there has been controversy about their actual localization. Once released extracellularly, these proteins are thought to act as damage-associated molecular patterns (DAMPs), though their mechanism of action is not well understood. These S100 proteins mainly form heterodimers (S100A8/A9, also known as calprotectin) and this heterocomplex is recognized as a useful biomarker for several inflammatory diseases. We observed that S100A8/A9 is highly present in the cytoplasmic fraction of neutrophils and is not part of the granule content. Furthermore, we found that S100A8/A9 was not released in parallel with granular content but upon the formation of neutrophil extracellular traps (NETs). Accordingly, neutrophils of patients with chronic granulomatous disease, who are deficient in phorbol 12-myristate 13-acetate (PMA)-induced NETosis, did not release S100A8/A9 upon PMA stimulation. Moreover, we purified S100A8/A9 from the cytoplasmic fraction of neutrophils and found that S100A8/A9 could induce neutrophil activation, including adhesion and CD11b upregulation, indicating that this DAMP might amplify neutrophil activation.

## 1. Introduction

The most abundant innate immune cells in the circulation consist of neutrophilic granulocytes. They circulate through the blood and are recruited to tissues during infection or inflammation. They are highly mobile, short-lived cells and comprise the first line of defense in the innate immune system [1]. When neutrophils are recruited to the site of infection, they recognize pathogen-associated molecular patterns (PAMPs) or damage-associated molecular patterns (DAMPs), which activate specific pattern recognition receptors (PRRs) on their surface. While PAMPs are derived from microbial molecules, DAMPs are endogenous molecules released during tissue damage [2]. Once activated, neutrophils are capable of eliminating pathogens by several mechanisms, i.e., phagocytosis, production of reactive oxygen species (ROS), degranulation, and the formation of neutrophil extracellular traps (NETs) [3].

The S100 family of proteins consists of 25 known members in humans [4]. These proteins are characterized by their EF-hand calcium-binding motif and are involved in numerous intracellular processes including proliferation, differentiation, and calcium homeostasis [5]. When present extracellularly, various S100s’ proteins can act as DAMPs. A subgroup of S100 proteins are calgranulins, consisting of calgranulin A (also known as S100A8 or myeloid-related protein-8 (MRP8)), calgranulin B (also known as S100A9 or MRP14), and calgranulin C (also known as S100A12). These proteins are particularly abundantly present in neutrophils, and S100A12 is even considered granulocyte specific [6]. S100A8 and S100A9 together comprise up to 40% of the cytosolic protein content of neutrophils [7], while approximately 8% of the cytosolic protein content consists of S100A12 [8,9]. These S100 proteins can form homodimers, but S100A8 and S100A9 mainly form non-covalently bound heterodimers (S100A8/A9, also known as calprotectin [10]) in the presence of calcium. Once released extracellularly, they are thought to act as DAMPs via Toll-like receptor 4 (TLR4) and the receptor for advanced glycation end products (RAGE) [11]. Furthermore, S100A8/A9 may be a useful biomarker for several inflammatory diseases like inflammatory bowel disease, various rheumatic diseases, fever syndromes, type 2 diabetes, and forms of vasculitis [12,13,14,15,16].

S100 proteins lack signal peptides required for the classical Golgi-mediated secretion pathway. Consequently, the release of S100A8/A9 is mediated by an alternative secretion pathway, which has not been fully elucidated [17]. As we and others have identified these proteins to be abundantly present within neutrophils [7,18], we studied these in more detail in the current study. There has been some controversy about the actual localization in neutrophils, i.e., S100A8/A9 is described to be present within the specific granules of neutrophils, while other groups describe S100A8/A9 to be present in the cytoplasm of neutrophils [7,19]. We found that S100A8/A9 is indeed highly present in the cytoplasmic fraction of neutrophils and is not part of the granule content. Using immunoelectron microscopy, we observed that S100A8 is primarily found in the cytoplasm as well as in granule membranes (but not in the granules). Additionally, we found that S100A8/A9 was released upon NETosis and not in parallel with granular content. Furthermore, we purified S100A8/A9 from the cytoplasmic fraction of neutrophils and showed that S100A8/A9 could induce neutrophil activation, including adhesion and CD11b upregulation, indicating that this DAMP might amplify neutrophil activation.

## 2. Materials and Methods

### 2.1. Isolation of Primary Neutrophils

Heparinized venous blood was drawn from healthy controls and chronic granulomatous disease (CGD) patients after informed consent had been obtained. Subsequently, neutrophils were isolated, as described previously [20], and resuspended in HEPES medium [21]. Cytospins were prepared and stained with May-Grünwald/Giemsa staining to evaluate neutrophil purity, morphology, and viability.

All experiments involving human blood samples were conducted in accordance with the Declaration of Helsinki. The study was approved by the local ethical committee of the Amsterdam University Medical Center and Sanquin Blood Supply, Amsterdam, The Netherlands.

### 2.2. Neutrophil Function Tests

#### 2.2.1. Neutrophil Degranulation

To assess degranulation of specific and azurophilic granules, neutrophils were pre-incubated with 1 µM of platelet-activating factor (PAF; Sigma-Aldrich, St Louis, MO, USA) or cytochalasin B (5 µg/mL; Sigma-Aldrich) for 5 min at 37 °C and subsequently stimulated for 10 min with formyl-Met-Leu-Phe (fMLF, 1 µM; Sigma-Aldrich). Protease levels were determined with DQ-BSA as previously described [22].

#### 2.2.2. Neutrophil Activation Markers

Neutrophils (1 × 10^5^) were incubated with tumor necrosis factor (TNF-α; 10 ng/mL Peprotech, London, UK) or the isolated S100A8/A9 fraction for 2 h at 37 °C. Flow cytometry was performed to assess the expression of neutrophil activation markers. Antibodies used were CD11b-FITC (mouse IgM clone CLB-mon-gran/1, B2, Sanquin reagents) and L-selectin-APC (mouse IgG clone DREG-56, BD Pharmingen, San Diego, CA, USA). Cells were analyzed on a FACSCanto-II flow cytometer (BD Biosciences, San Jose, CA, USA). Neutrophils were gated based on their forward- and side-scatter, and 10,000 gated events per sample were collected. Data are expressed as mean fluorescence intensity (MFI) and are corrected for relevant isotype controls.

#### 2.2.3. Neutrophil Apoptosis Measurement

Neutrophils (1 × 10^5^) were incubated with the isolated S100A8/A9 fraction for 2 h at 37 °C. Neutrophil viability was assessed by flow cytometry with Annexin V-Fluorescein isothiocyanate (FITC, dilution 1:300) (BD Biosciences) and TO-PRO-3 stain, dilution 1:5000 (Thermo Fisher Scientific, Waltham, MA, USA), as previously described [23]. Viable cells were defined as negative for Annexin V-FITC and TO-PRO-3.

#### 2.2.4. Neutrophil Adhesion and Priming of fMLF-Induced Oxidative Activity

Neutrophil adhesion and priming in response to S100A8/A9 were assessed, as previously described [22,23]. In brief, for adhesion experiments, neutrophils were labeled with calcein-AM (1 μM; Molecular Probes, Eugene, OR, USA) and stimulated with either lipopolysaccharide (LPS) and LBP (LPS-binding protein), the S100A8/A9 fraction, or left unstimulated and incubated for 30 min at 37 °C in an uncoated 96-well MaxiSorp plate (Nunc, Wiesbaden, Germany). Subsequently, plates were washed three times with PBS. Adherent cells were lysed in 0.5% (*w*/*v*) Triton X-100 in PBS and fluorescence was measured with an Infinite F200 PRO plate reader (Tecan, Männedorf, Switzerland) at an excitation wavelength of 485 nm and an emission wavelength of 535 nm. Adhesion was determined as percentage of total input of calcein-labeled cells [22].

In brief, for priming experiments, neutrophils were pre-incubated with either LPS and LBP, the S100A8/A9 fraction, or left unstimulated for 30 min at 37 °C before stimulation with fMLF (1 µM). NADPH-oxidase activity was measured as the release of hydrogen peroxide (H_2_O_2_) with an Amplex Red kit (Molecular Probes). Fluorescence (excitation 535 nm; emission 595 nm) was monitored at 30-s intervals for 30 min by an Infinite F200 PRO plate reader. Data are expressed as the maximal slope of H_2_O_2_ release per 2-min interval [23].

#### 2.2.5. Neutrophil Extracellular Traps’ Imaging and DNA Release Measurements

Neutrophils (2 × 10^5^) were seeded on 12-mm glass coverslips and NET formation was induced with phorbol 12-myristate 13-acetate (PMA; 100 ng/mL, Sigma-Aldrich) or MSU crystals (200 µg/mL, Invivogen, San Diego, CA, USA) for 4 h at 37 °C. Subsequently, cells were stained with primary antibodies directed against S100A8/A9 (0.5 µg/mL) (mouse monoclonal, clone 27E10, BMA Biomedicals, Augst, Switzerland) or myeloperoxidase (MPO, 2 µg/mL) (mouse monoclonal [2C7], Abcam, Cambridge, UK) and neutrophil elastase (3 µg/mL) (rabbit polyclonal, Sanquin Reagents, Amsterdam, The Netherlands). The secondary antibodies used were F(ab’)2-Goat anti-Mouse IgG (H + L) Alexa Fluor 633 (4 µg/mL) and F(ab’)2-Goat anti-Rabbit IgG (H + L) Alexa Fluor 488 (4 µg/mL). DNA was stained by Hoechst 33342 and coverslips were imaged on a Leica TCS SP8 confocal microscope and analyzed with Leica Application Suite X (Leica Microsystems, Wetzlar, Germany).

Quantification of DNA release was measured with Sytox Green Nucleic Acid Stain (Thermo Fisher Scientific). Neutrophils were seeded in a 96-well white microplate (Corning Inc., Corning, NY, USA) and stained with Sytox Green (1 µM). NET formation was induced with PMA (100 ng/mL) and mono-sodium urate (MSU) crystals (200 µg/mL). Fluorescence (excitation 485 nm; emission 535 nm) was monitored at 1-min intervals for 4 h by an Infinite F200 PRO plate reader (Tecan, Männedorf, Switzerland) at 37 °C. Data are expressed as relative fluorescence units (RFU).

### 2.3. S100 Protein Biochemistry, Release, and Microscopy

#### 2.3.1. ELISA

S100A8/A9 and lactoferrin were measured by in-house-developed, enzyme-linked immunosorbent (ELISA) assays, as previously described [15,24]. In short, 96-well, flat-bottom plates were coated with primary antibodies directed against S100A8/A9 (0.5 µg/mL, mouse monoclonal, clone 27E10, BMA Biomedicals) or lactoferrin (1 µg/mL, mouse monoclonal, M9160, Sanquin reagents). Supernatants from stimulated neutrophils were incubated for 1 hour at room temperature. Biotinylated monoclonal mouse IgG1 S100A9 (0.5 µg/mL, clone S36.48, BMA Biomedicals) or biotinylated polyclonal anti-lactoferrin antibodies (0.25 µg/mL, Sanquin reagents) were used as detection antibody. After 1 hour of incubation, plates were washed and streptavidin conjugated to (poly-)horseradish peroxidase was added followed by 0.1 mg/mL tetramethylbenzidine (TMB; Merck Chemicals) in 0.11 M sodium acetate, with 0.003% (*v*/*v*) H_2_O_2_. The reaction was stopped with H_2_SO_4_. Absorbance was measured at 450/540 nm and results are expressed in µg/mL by reference of the calibration curve.

#### 2.3.2. Isolation of Cytosolic Fraction of Neutrophils

The cytosolic fraction of neutrophils was isolated using the glycoside digitonin. Neutrophils (50 × 10^6^ cells/mL) were resuspended in ice-cold PBS containing 150 µM of digitonin (Merck, Darmstadt, Germany). After 10 min of incubation at 4 °C, the suspension was centrifuged at 20,000× *g* and supernatant containing the cytosolic fraction was harvested. Successful cytosol isolation by digitonin was determined by measurement of lactate dehydrogenase (LDH) levels and absence of proteases in the fraction. Protease levels were determined with DQ-Green BSA (Molecular Probes, Eugene, OR, USA). Absence of DNA in cytosolic fraction was confirmed with Sytox Green Nucleic Acid Stain. The cytosolic fraction was stored at −80 °C if not used immediately.

#### 2.3.3. Isolation and Purification of S100A8, S100A9, and S100A8/A9

For the isolation of S100A8, S100A9, and hetero/homo-complexes from the cytosolic fraction, we adapted a previously described method [25]. In short, 50% (*w*/*v*) ammonium sulphate (AS) was added to the cytosolic fraction and incubated on ice for 1 hour. After centrifugation at 10,000× *g* for 30 min at 4 °C, supernatant was dialyzed using a 3.5 K MWCO Slide-A-Lyzer Dialysis Cassette (Thermo Fisher Scientific, Waltham, MA, USA) against “buffer A” (1 mM EDTA and 50 mM Tris-HCl in demi water, pH 8.5) at 4 °C. During the first and second hours of dialysis, buffer A contained 25% and 12.5% (*w*/*v*) AS, respectively, before dialyzing against 0% (*w*/*v*) AS overnight.

Anion exchange chromatography (AEX) was performed using the ÄKTA avant (GE Healthcare, Chicago, IL, USA) and a 1-mL HiTrap MonoQ AEX column (GE Healthcare). A salt gradient of 0 to 1 M with buffer A and “buffer B” (buffer A + 1 M NaCl) in 20-column volumes (CVs) was used to elute proteins. Fractions containing at least 100 µg/mL of S100A8/A9 (as determined by ELISA detecting the S100A8/A9 hetero-complex) were pooled and dialyzed against PBS using a 3.5 K MWCO Slide-A-Lyzer Dialysis Cassette to remove buffer A.

#### 2.3.4. SDS-PAGE and Western Blot Analysis

Samples were separated by SDS polyacrylamide gel electrophoresis and transferred onto a nitrocellulose membrane for Western Blotting or gels were stained with InstantBlue Coomassie protein stain (Expedeon, San Diego, CA, USA) to visualize all proteins. Individual proteins were detected with antibodies against S100A8 (1.5 µg/mL) (MAB4570, mouse monoclonal, R&D systems, Minneapolis, MN, USA) or S100A9 (dilution 1:1000) (MBS2540112, rabbit polyclonal, MyBioSource, San Diego, CA, USA).

Secondary antibodies were goat anti-mouse-IgG IRDye 680RD (0.2 µg/mL), goat anti-rabbit-IgG IRDye 800CW (LI-COR Biosciences, Lincoln, NB, USA) (0.2 µg/mL), HRP-conjugated rabbit anti-mouse (Sanquin Reagents) (dilution 1:1000), or HRP-conjugated horse anti-rabbit (dilution 1:250) (Sanquin Reagents). Quantification of bound antibodies was performed on an Odyssey Infrared Imaging system (LI-COR Biosciences) or proteins were visualized using Pierce ECL Western Blotting Substrate (Thermo Fisher Scientific) and the Universal Hood II (Bio-Rad laboratories, Hercules, CA, USA).

#### 2.3.5. Immuno-Electron Microscopy Analysis

For electron microscopy, purified neutrophils were immuno-labelled, as previously described [26]. Sections were incubated with a monoclonal mouse anti-S100A8 antibody (5 µg/mL) (MAB4570, R&D systems) followed by a bridging antibody rabbit anti-mouse IgG (Nordic) and 10-nm protein-A conjugated colloidal gold (EMlab, University of Utrecht, The Netherlands) and examined with a Tecnai12G2 electron microscope (Thermo Fisher, Eindhoven, The Netherlands).

### 2.4. Statistics

Experimental data were plotted and analyzed by GraphPad Prism V8.0.2 (GraphPad Software, San Diego, CA, USA). Results are shown as mean ± standard error of mean (SEM). The paired t-test or mixed effects model with Dunnett post hoc test was used to test statistical significance (* = *p* < 0.05; ** = *p* < 0.01; *** = *p* < 0.001; **** = *p* < 0.0001; ns = non-significant).

## 3. Results

### 3.1. S100A8/A9 Is Not Released in Parallel with Granular Contents during Exocytosis

**Figure 1:**
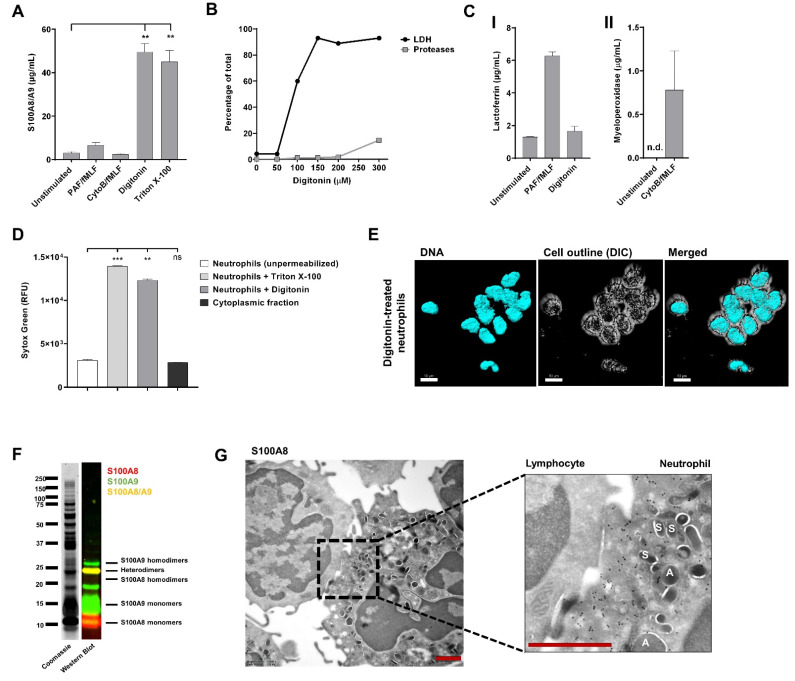
S100A8/A9 is not released during exocytosis. (**A**) Neutrophils (5 × 10^5^) from healthy controls were left unstimulated or incubated with PAF (1 µM, 5 min) or cytochalasin B (5 µg/mL, 5 min) and subsequently stimulated with fMLF (1 µM) for 10 min at 37 °C. To obtain the cytoplasmic fraction of neutrophils, digitonin (150 µM, 15 min) was used to permeabilize the plasma membrane of neutrophils. As a positive control, neutrophils were permeabilized with Triton X-100 (1%, 10 min). S100A8/A9 concentration was determined by ELISA (n = 4–6, mean + SEM). (**B**) Experiment to determine the optimal concentration of digitonin to isolate the cytoplasmic fraction. Lactate dehydrogenase (LDH) levels and protease levels were measured and are presented as percentage of their total content (n = 1). (**C**) Panel I: Lactoferrin concentration in supernatant of unstimulated, PAF/fMLF-stimulated neutrophils, or the cytoplasmic fraction of neutrophils was determined by ELISA (n = 2, mean + SEM). Panel II: Myeloperoxidase concentration in supernatant of unstimulated or CytoB/fMLF-stimulated neutrophils was determined by ELISA (n = 2, mean + SEM). (**D**) Presence of DNA was measured with Sytox Green Nucleic Acid Stain (n = 3, mean + SEM). (**E**) Representative image of digitonin-treated neutrophils. DNA was visualized with Hoechst (blue, 405). Images were acquired using a Leica SP8 confocal microscope, magnification 630x, scale bar = 10 µm. (**F**) Coomassie Brilliant Blue staining and Western Blot for S100A8 (red) and S100A9 (green) of the cytoplasmic fraction of neutrophils. (**G**) Representative immuno-electron microscopy image of a neutrophil (right) and lymphocyte (left) stained for anti-S100A8 (black dots). Scale bar = 1 µm. “A” indicates azurophilic granules and “S” indicates specific granules. S100A8 is not present in neutrophil granules. Statistics were performed by mixed effects’ model (**A**) or one-way ANOVA (**D)** with Dunnett post hoc test; ** *p* < 0.01, *** *p* < 0.001, ns = not significant; n.d. = not detectable.

While S100A8/A9 lacks signal peptides required for secretion via the classical ER/Golgi route [17], it has been suggested that S100A8/A9 is localized within the specific granules of neutrophils and that stimulation of specific granule exocytosis resulted in the release of S100A8/A9 [19]. However, it has also been suggested to be present in the cytoplasm of neutrophils [7]. Therefore, we prepared supernatants from activated neutrophils and measured S100A8/A9 levels by ELISA to determine their localization (Figure 1A). When exocytosis of specific granules was induced with platelet-activating factor (PAF) in combination with fMLF (a bacterial chemotactic peptide), no increase in S100A8/A9 release was observed. Similar results were obtained when inducing the release of the specific and azurophilic granules with fMLF, following pre-incubation with cytochalasin B (CytoB; actin polymerization inhibitor). Specific granule exocytosis and azurophilic granule exocytosis were confirmed by measurement of lactoferrin and myeloperoxidase, respectively (Figure 1C).

As S100A8/A9 was not found to be released upon neutrophil degranulation, we investigated if the complex was present in the cytoplasm of neutrophils. Therefore, we isolated the cytosolic fraction of neutrophils using the glycoside digitonin. This compound allows for the selective permeabilization of the plasma membrane, leaving intracellular membrane-bound organelles (e.g., granule membranes) intact [27]. Successful isolation of cytosol was determined by the presence of lactate dehydrogenase (LDH) and absence of proteases in the fraction (Figure 1B). As expected, release of the cytosolic fraction by digitonin treatment did not result in increased lactoferrin levels compared to supernatants of unstimulated neutrophils, confirming that digitonin treatment did not disturb the granule membranes (Figure 1C). Furthermore, we confirmed that permeabilization with digitonin did not lead to leakage of DNA in the cytoplasmic fraction (Figure 1D). However, the nuclear morphology of neutrophils did not remain fully segmented, indicating that digitonin has an effect on the nuclear membrane (Figure 1E). High levels of S100A8/A9 were detected in the cytosolic fraction of neutrophils, which was comparable to the levels found in whole neutrophil lysates (Triton-X 100 condition; Figure 1A). Coomassie Brilliant Blue staining and Western Blot for S100A8 and S100A9 after SDS-PAGE confirmed the presence of the heterodimer S100A8/A9 in the cytoplasmic fraction and also showed high levels of S100A8 and S100A9 monomers and, to a lower extent, homodimers (Figure 1F).

To confirm the localization of S100A8 in neutrophils, we performed immunoelectron microscopy. We showed that S100A8 was primarily found in the cytoplasm but also in granule membranes (but not in granules) and in the nucleus, where it mostly localized in euchromatin regions (Figure 1G). Lymphocytes in the same preparations were negative for S100A8, which suggests a lack of false-positive nuclear background staining. Localization of S100A8/A9 within the nucleus was also previously observed by Urban et al. [28] and can be explained by passive diffusion of small proteins (<40–60 kDa) through the nuclear pore complex [29].

Taken together, our results indicate that the S100A8/A9 heterodimers are not released in parallel with granular contents during exocytosis.

### 3.2. S100A8/A9 Is Released during NETosis and Not via Degranulation

When neutrophils become strongly activated, these cells can release their DNA content and form so-called NETs. These NETs are web-like chromatin structures, covered with histones, granule-derived proteases, and antimicrobial peptides [30]. During this process of cell death, which is known as NETosis, the intracellular content of neutrophils is concomitantly released and, thus, may act as an alternative secretion pathway in a relatively controlled manner [31].

**Figure 2:**
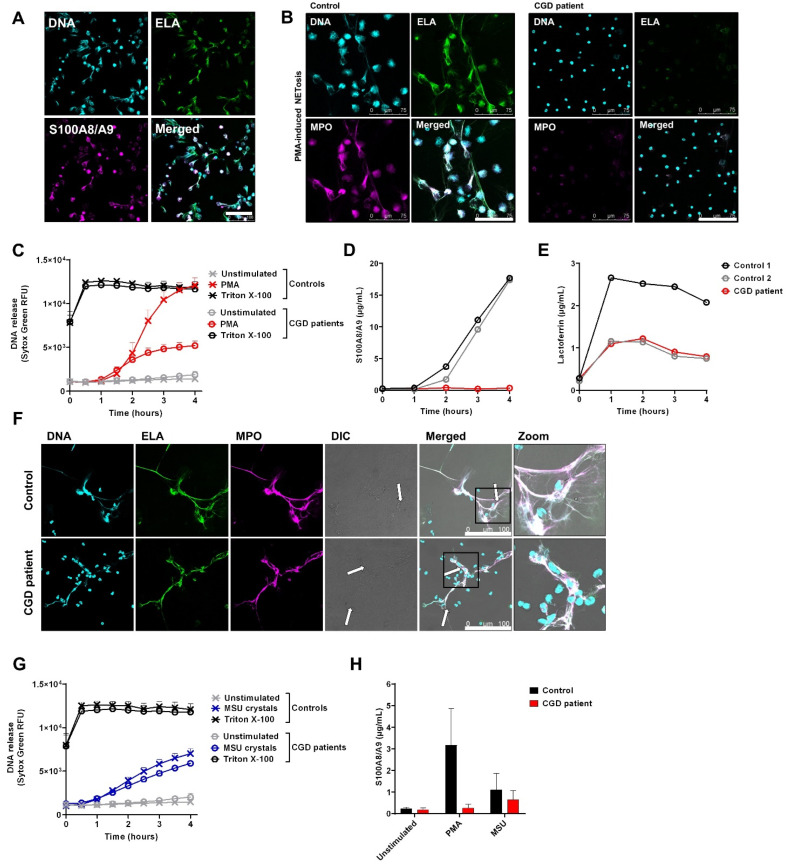
S100A8/A9 release by neutrophils upon NETosis. To induce NET formation, neutrophils were stimulated with (**A**–**E**) PMA (100 ng/mL) or (**F**–**H**) MSU crystals (200 µg/mL) for 4 h at 37 °C. (**A**,**B**,**F**) NETs were visualized by staining for Elastase (ELA) (green, 488), DNA (Hoechst, blue, 405), and (**A**) S100A8/A9 or (**B**,**F**) myeloperoxidase (MPO) (magenta, 633). Images were acquired using a Leica SP8 confocal microscope, magnification 400×. Scale bar = (**A**,**F**) 100 µm or (**B**) 75 µm. (**C**,**G**) DNA release of neutrophils was measured by Sytox Green Nucleic Acid Stain upon stimulation with (**C**) PMA (n = 8 for controls and n = 4 unique CGD patients, of which one patient was measured three times, mean + SEM) or (**G**) MSU crystals (n = 5 for controls and n = 3 unique CGD patients, of which one patient was measured two times, mean + SEM). Triton X-100 was used as positive control. (**D**,**E**) Supernatant of PMA-stimulated neutrophils was collected at the indicated time points and measured for (**D**) S100A8/A9 and (**E**) lactoferrin by ELISA. (**F**) Arrows indicate NET structures around MSU crystals. (**H**) Supernatants of neutrophils stimulated for 4 h with PMA or MSU crystals were collected and measured for S100A8/A9 by ELISA (n = 2 for controls, n = 2 for CGD patients, mean + SEM). CGD = chronic granulomatous disease.

To induce NET formation, neutrophils are classically stimulated with phorbol 12-myristate 13-acetate (PMA) for 4 h at 37 °C. NETs were visualized by staining for DNA. As also previously shown [28], we could confirm the presence of S100A8/A9 in these NETs (Figure 2A). This indicates that S100A8/A9 may be co-released during NETosis, similar to granular content, as indicated by the positive staining of elastase (Figure 2A) or MPO (data not shown). When investigating neutrophils that are unable to release NETs (i.e., neutrophils from patients with chronic granulomatous disease (CGD) unable to generate ROS), we observed a strongly diminished DNA release upon PMA treatment, corresponding to impaired ROS-dependent NETosis by these patient cells (Figure 2B,C) [32]. Furthermore, there was also no S100A8/A9 release observed (Figure 2D). In contrast, when NETosis is induced in healthy control neutrophils, S100A8/A9 is simultaneously released with DNA (Figure 2C,D). Release of lactoferrin from the specific granule showed completely different kinetics, i.e., the peak of lactoferrin levels in the supernatant was reached even before DNA (and thus NETs) was released, again indicating that S100A8/A9 is not released in parallel with specific granule proteins (Figure 2E).

As PMA is a very potent but non-physiological agent, we also induced NET formation by mono-sodium urate (MSU) crystals as found in gout as an inflammatory trigger. Of note, MSU crystals induce NETosis in a ROS-independent manner [33], indicating that CGD neutrophils are able to form NETs per se (Figure 2F,G). Again, we observed that S100A8/A9 is released in conditions when NETs are formed (Figure 2H).

### 3.3. Isolation and Purification of S100A8/A9 from Neutrophils

**Figure 3:**
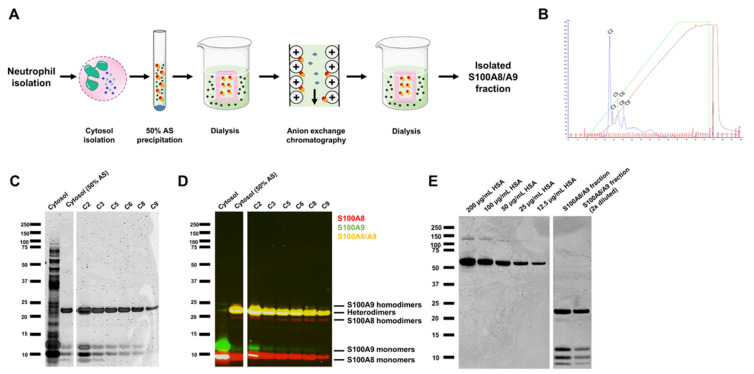
S100A8/A9 purification of the cytoplasm of neutrophils. (**A**) Schematic overview of the purification process of S100A8/A9 from neutrophil cytosol. (**B**) The cytoplasmic fraction of neutrophils was isolated followed by 50% ammonium sulfate (AS) precipitation and anion-exchange chromatography (AEX). Indicated are the different peaks that were isolated (C2–C9). (**C**) Coomassie Brilliant Blue staining and (**D**) Western blot of isolated peak fractions, identifying S100A8 (red) and S100A9 (green) to be presented as mostly heterodimers and monomers. (**E**) Coomassie Brilliant Blue staining with a human serum albumin (HSA) calibration curve that was used to calculate the concentration of S100A8 and A9 heterodimers and monomers in the S100A8/A9 fraction used for functional experiments.

To clarify the subsequent inflammation-propagating activity of S100A8/A9 proteins once released as local or systemic activating signals for neutrophils themselves, we purified S100A8/A9 from the cytoplasmic fraction of neutrophils using digitonin. As previously mentioned, digitonin does not disturb the granule membranes [27], thereby preventing release of proteases, which can cleave proteins of interest. Upon isolation of the cytoplasmic fraction, a 50% ammonium sulphate precipitation and anion-exchange chromatography (AEX) were followed (Figure 3A,B). Coomassie staining and Western blot of isolated AEX peak fractions showed that S100A8 (10.8 kDa) and S100A9 (13.2 kDa) proteins were present as monomers and, most abundantly, heterodimers. Homodimers were only faintly visible (Figure 3C,D). AEX peak fractions containing >100 µg/mL S100A8/A9 as measured by ELISA were pooled and dialyzed to PBS. Subsequently, a human serum albumin (HSA) calibration curve was used to calculate the concentration of S100A8 and A9 heterodimers and monomers in the S100A8/A9 fraction used for functional experiments (Figure 3E). The concentration of the heterodimer S100A8/A9 was 90 µg/mL, and 8 and 9 µg/mL for the monomers S100A8 and A9, respectively. We identified the protein <10 kDa in our fraction as S100A12 (data not shown). Next, the S100A8/A9 fraction was used for functional experiments on neutrophils.

### 3.4. S100A8/A9 Induces Neutrophil Activation

**Figure 4:**
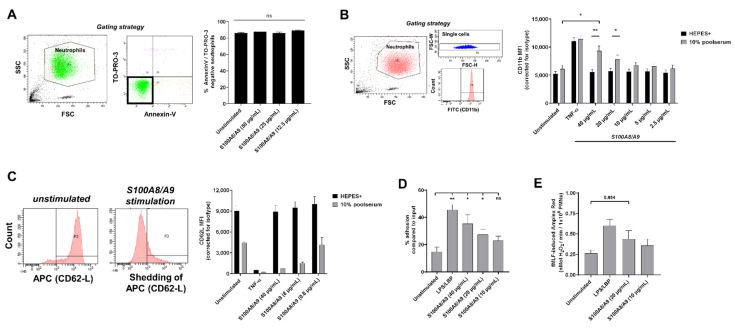
S100A8/A9 activates neutrophils. (**A**) Viability of neutrophils upon 2 h of incubation at 37 °C with S100A8/A9 was assessed by flow cytometry for AnnexinV and TO-PRO. Viable neutrophils were defined as negative for both markers (n = 3, mean + SEM). Indicated in the left panel is the gating strategy. (**B**,**C**) Expression of neutrophil activation markers (**B**) CD11b (n = 3–5, mean + SEM) and (**C**) CD62L (n = 2, mean + SEM) was measured by flow cytometry upon 2 h of incubation at 37 °C with indicated concentrations of S100A8/A9 or TNF-α in the absence or presence of 10% human serum. (**B**) Indicated in the left panel is the gating strategy. (**C**) FACS plots showing CD62-L shedding are displayed. (**D**) Neutrophils were left unstimulated or stimulated with LPS/LBP or different concentrations of S100A8/A9 for 30 min at 37 °C in the presence of 10% human serum. Percentage of adhesion is shown compared to total input (n = 3–5, mean + SEM). (**E**) Neutrophils were primed by LPS/LBP or different concentrations of S100A8/A9 in the presence of 10% human serum. After 30 min of incubation at 37 °C, 1 µM fMLF was added to induce ROS production. The maximal slope of H_2_O_2_ production was measured using Amplex Red (n = 5, mean + SEM). Statistics were performed by paired t-test (**A**,**B**,**E**) or mixed effects’ model with Dunnett post hoc test (**D**); * *p* < 0.05, ** *p* < 0.01, ns = not significant.

Before we assessed neutrophil activation by S100A8/A9, we ascertained it was not cytotoxic for the cells (Figure 4A). To investigate if S100A8/A9 induced neutrophil activation, we measured upregulation of the neutrophil activation marker CD11b by flow cytometry. While S100A8/A9 alone did not induce upregulation of CD11b, the addition of 10% human serum did lead to CD11b upregulation to a similar extent as tumor necrosis factor-α (Figure 4B). In the presence of 10% human serum, S100A8/A9 induced neutrophil activation in a dose-dependent manner. Furthermore, shedding of CD62L (L-selectin), another neutrophil activation marker, showed similar results (Figure 4C). Therefore, we continued our experiments with the addition of 10% human serum.

We assessed the adhesion and priming capacity of neutrophils upon incubation with S100A8/A9. As control, we also stimulated neutrophils with lipopolysaccharide (LPS) and LBP (LPS-binding protein), which activates TLR4, the same receptor that is thought to be activated by S100A8/A9. Stimulation with LPS/LBP or S100A8/A9 resulted in an increase in neutrophil adhesion, and S100A8/A9 again showed a dose-dependent response (Figure 4D). Priming is the transition of neutrophils from a basal state towards an enhanced responsiveness state [34] and can be determined by measuring increased fMLF-induced ROS production by neutrophils upon pre-incubation with a priming agent. Upon pre-incubation with S100A8/A9, neutrophils showed an enhanced ROS production when stimulated with fMLF, indicating S100A8/A9 is capable of priming neutrophils (Figure 4E).

In conclusion, S100A8/A9 induces neutrophil activation, including adhesion, CD11b upregulation, and shedding of CD62L, indicating that this DAMP might amplify neutrophil activation.

## 4. Discussion

S100A8/A9 is recognized as a useful biomarker for several inflammatory diseases and is thought to act as DAMP once released extracellularly. However, contradictory reports are published about the mechanism by which S100A8/A9 is released. Here, we showed that S100A8/A9 is not released during exocytosis of specific and azurophilic granules but during NETosis.

We observed the presence of S100A8/A9 in NETs by immunocytochemistry and showed that the release of S100A8/A9 occurred simultaneously with the release of DNA during the process of NETosis, i.e., after 1.5–2 h of neutrophil activation. This was in contrast to lactoferrin, a specific granule component and another constituent of NETs, which reached their maximum concentration even before DNA (and thus NETs) were released. This indicates that release of lactoferrin occurs via degranulation and it is, therefore, reasonable that positively charged lactoferrin [35] binds extracellularly to NETs, i.e., upon release of negatively charged DNA by neutrophils. During NETosis, chromatin decondensation is induced, whereupon nuclear material mixes with the cytoplasm, which ultimately leads to plasma membrane rupture and release of NETs [32]. Whether S100A8/A9 initially binds to DNA intracellularly when the nuclear material mixes with the cytoplasm or if it binds to the NETs subsequently to DNA release remains to be determined. Nevertheless, S100A8/A9 seems to be a reliable marker to monitor NETosis kinetics in vitro.

For several inflammatory diseases (e.g., inflammatory bowel disease, various rheumatic diseases, fever syndromes, and forms of vasculitis) S100A8/A9 is recognized to be a useful biomarker [12,13,14,15]. NETs are indicated to be involved in the pathophysiology of the former indicated inflammatory disorders and NET degradation products, such as circulating free DNA or elastase-DNA and MPO-DNA complexes, found in higher levels in serum or plasma of these patients [36,37,38,39]. As we showed that S100A8/A9 is specifically released during NETosis, it is, therefore, likely that high serum levels of S100A8/A9 found in these patients is due to dysregulated NETosis. However, circulating free DNA and S100A8/A9 might also be derived from necrotic cells and, therefore, the exact contribution of NETosis needs to be determined.

We showed in vitro that S100A8/A9 can activate neutrophils themselves, leading to induction of neutrophil activation. However, we only observed neutrophil activation by S100A8/A9 in the presence of human serum. This might suggest that a component in serum is needed to convert S100A8/A9 to an active state or that there is synergism with an as of yet unidentified serum-derived protein to induce activation. It was previously found that S100A8/A9 can enhance inflammatory responses by inducing cytokine secretion by PBMCs and activating β2 integrin activation in neutrophils [40,41,42], though it also has been suggested that calcium-dependent tetramerization of S100A8/A9 restricts its inflammatory activity [43]. However, according to Vogl et al. [43], 100 µM calcium ions were sufficient to induce tetramer formation of S100A8/A9 complexes and, thus, to abolish inflammatory activity, while we and others performed experiments in the presence of (>100 µM) calcium and observed pro-inflammatory activity of S100A8/A9 [40,42].

Overall, we demonstrated that neutrophils release S100A8/A9 via NETosis and that neutrophils themselves are activated by S100A8/A9, indicating that this DAMP induces neutrophil activation. There is a recent interest in NETs as potential therapeutic targets for several inflammatory disorders [44,45], and successful NET inhibition might, thus, also prevent S100A8/A9-induced inflammation.

## Data Availability

The data that support the findings of this study are available from the corresponding author, E.G.G.S., upon request.
